# Unmasking Gastroparesis in Diabetes During Ramadan: Challenges and Management Strategies

**DOI:** 10.3390/jcm14061997

**Published:** 2025-03-15

**Authors:** Mohammed Abdulrasak, Nael Shaat, Ali M. Someili, Mostafa Mohrag

**Affiliations:** 1 Department of Clinical Sciences, Lund University, 22100 Malmo, Sweden; nael.shaat@med.lu.se; 2 Department of Gastroenterology and Nutrition, Skane University Hospital, 21428 Malmo, Sweden; 3 Department of Endocrinology, Skåne University Hospital, 21428 Malmo, Sweden; 4 Department of Medicine, Faculty of Medicine, Jazan University, Jazan 45142, Saudi Arabia; ali.someili@medportal.ca (A.M.S.); mmohrag@jazanu.edu.sa (M.M.)

**Keywords:** type 2 diabetes, diabetes, Ramadan, fasting, gastroparesis, GLP-1, gastric emptying

## Abstract

Gastroparesis, characterized by delayed gastric emptying without mechanical obstruction, is a recognized complication of long-standing diabetes. Its pathophysiology involves, amongst other mechanisms, autonomic dysfunction due to vagal nerve damage, impaired smooth muscle contractility, and hormonal dysregulation of intestinal motility. During Ramadan, fasting causes significant dietary changes due to prolonged fasting and the consumption of large meals for Iftar (breaking of fast), which may unmask or worsen gastroparesis symptoms in individuals with diabetes. Symptoms such as early satiety, bloating, nausea, and glycemic fluctuations can further complicate diabetes management during fasting. This paper highlights the relationship between Ramadan fasting and gastroparesis in individuals with diabetes, exploring underlying mechanisms, clinical manifestations, diagnostic approaches, and management strategies. A multidisciplinary approach involving dietary modifications, medication adjustments, lifestyle changes, and individualized medical counseling is essential for safe fasting, alongside the option to avoid fasting in individuals who are deemed too high at risk for fasting. Further research is needed to assess the prevalence of subclinical gastroparesis in fasting individuals with diabetes and to optimize risk stratification and management in these patients.

## 1. Introduction

Gastroparesis involves the delayed emptying of gastric contents into the duodenum in the absence of a mechanical obstructive process [1]. The diagnosis is a well-recognized complication of diabetes, particularly in individuals with a disease duration of over 10 years and concurrent complications [2]. The postulated mechanism causing gastroparesis involves injury to the vagus nerve with ensuing poor autonomic control of the stomachs’ motility, albeit underlying diabetes control is also a contributing factor [3,4]. This complication is more common in individuals suffering from type 1 (T1D) than type 2 diabetes (T2D), with the spectrum of symptoms ranging from completely asymptomatic to having severe symptoms of early satiety, nausea and vomiting, or other gastrointestinal symptoms [5].

Muslims who are healthy are obligated by their religion to fast the month of Ramadan, during which no food or drink is consumed from sunrise (Sahoor) to sunset (Maghrib) [6]. Patients with well-controlled T2D without complications can fast if they wish to do so, while those with T1D or T2D with complications are advised against fasting [7]. This pattern of fasting may cause significant alterations in the dietary habits of fasting individuals, from eating regular meals at set intervals to larger quantities of food after a prolonged period of fasting. Such large meals have been shown to worsen gastroparesis symptoms [8,9]. It is, therefore, likely that such an eating pattern may unmask undiagnosed gastroparesis in individuals with diabetes. Roughly 115–150 million Muslims are thought to be diabetic, with the number most likely being a gross underestimation [10,11].

Furthermore, the prevalence of gastroparesis in the diabetic population is estimated at roughly 1–9% overall depending on the population at hand and the underlying type of diabetes in question, with increased prevalence in patients with prolonged duration—especially if diagnosed for 10 years or more—with underlying diabetes and a slightly increased prevalence in females compared to males [12,13,14]. There is a clear predisposition for those with T1D for developing this condition, as previously mentioned [12]. Regional differences are challenging to discern given the paucity of studies from non-western countries alongside the heterogeneity of the available publications both with regard to the sample size of patients included alongside the methods with which gastroparesis was diagnosed. In spite of this, a systematic review and meta-analysis analyzing the presence of gastroparesis in diabetes [13] revealed a large difference in prevalence between different regions. For instance, based on available studies, the prevalence level of 6–10% was reported in Saudi Arabia [15,16,17], while a prevalence of 6–41% was present in China [13] and 1–5% in the USA [13]. The majority of these studies were published, however, prior to the wide adoption of GLP-1 (glucagon-like peptide-1) analogs as a cornerstone in the management of T2D, which may further represent a gross representation of the reality of gastroparesis in individuals with diabetes [18]. The diagnosis is associated with a significant increase in healthcare utilization amongst the diabetic population, and this will likely increase based on the longer life expectancies of these individuals and increased GLP-1 use which further exacerbates the condition [19].

This review aims to highlight the relationship between Ramadan fasting and the occurrence of gastroparesis symptoms in individuals with diabetes, delving into pathophysiological mechanism, clinical symptoms, and management strategies.

## 2. Gastroparesis in Diabetes—Pathophysiological Considerations

In normal individuals, the main function of the stomach is to accommodate for the storage of food and fragment solid food into smaller portions which are easier to absorb by the duodenum. Swallowing triggers the relaxation of the gastric fundus to help attain a larger volume of food; thereafter, increased pressure in the fundus portion of the stomach occurs in transferring contents from the funds to the antrum. In the antrum, a to-and-fro motion occurs such that solid food is “mixed together” with the gastric juices to form a liquid “soup” called chyme [20]. Deficit in any of the portions of this process may cause gastroparesis.

**Figure 1:**
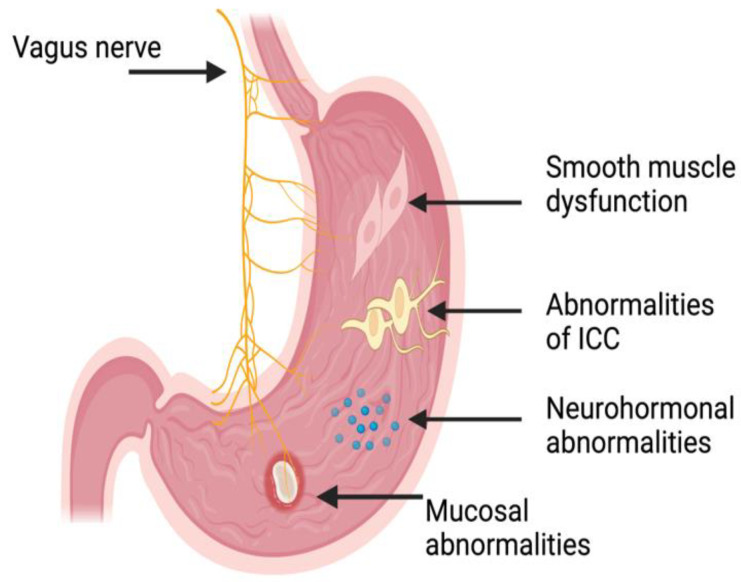
Illustration (not to scale) of the potential deficits which may lead to gastroparesis and/or its symptoms and the anatomical locations of these. ICC: Intestinal cells of Cajal. Illustration generated using BioRender™.

Multiple factors (summarized in Figure 1) come into play with regard to gastroparesis in the diabetic population. The main mechanism behind this is the development of the *autonomic dysfunction*, mainly with regard to the vagus nerve, in patients with long-standing diabetes [21]. The vagus nerve is important for coordinating the different parts of the gastric emptying (GE) “symphony”, as it regulates the contraction and relaxation of the different portions of the smooth musculature of the stomach [22]. Injury to the vagus nerve due to long-standing diabetes, therefore, disrupts normal gastric peristalsis, causing hindered GE, which leads to bloating sensation, nausea, and vomiting, all of which are classical gastroparesis symptoms [21,22].

The “maestro” with regard to the GE “symphony” may be the interstitial cells of Cajal (ICC), also known as the pacemaker cells of the stomach. These cells are specialized cells in the myenteric plexus of the stomach, and they undergo spontaneous depolarization–repolarization cycles whereby the smooth muscles of the stomach undergo rhythmic contractions [23,24]. Previous studies demonstrated that patients with diabetes undergo *a loss of these specialized (i.e., ICC) cells*, which, in effect, causes an irregular and uncoordinated contraction of the smooth muscles of the stomach [25,26]. In addition to this, the ICC is affected by increased oxidative stress both directly through mitochondrial dysfunction and indirectly due to the lack of antioxidant-supporting macrophages, which are essential for ICC function and longevity [27,28,29]. This, in effect, leads to worsened gastric emptying and an aberrant pattern of motility in the stomach—with antral hypomotility and pyloric spasm—but also in the entire gastrointestinal tract [25,26].

The effector that induces motility, i.e., the smooth-muscle cells (SMCs) in the stomach, are also afflicted by underlying diabetes. This occurs due to multiple mechanisms, including increased oxidative stress induced by hyperglycemia, leading to chronic inflammation mainly through a macrophage response, which, at the microscopic level, leads to *damaged SMCs* and, therefore, a loss of function [30,31,32]. Additionally, the SMCs and their surrounding extracellular matrix undergo remodeling in states of high oxidative stress, which subsequently impairs SMC function and further contributes to reduced gastric motility [33,34]. The aforementioned will, in effect, diminish the contractile apparatus of the stomach and further worsen gastroparesis.

Apart from the neural control exerted by the vagus nerve and the muscular effect through the stomach SMCs, the gastrointestinal hormones play an important role with regard to gastroparesis [35]. Motilin and Ghrelin are the main hormones promoting GE, while GLP-1 and CCK (cholecystokinin) are the main ones inhibiting GE [36]. The hormonal control of GE is aberrant in diabetic patients; for instance, *ghrelin levels are lower* in certain individuals with diabetes, which may contribute to the pathogenesis of poor GE in these patients [37,38].

As briefly mentioned in previous literature, *hyperglycemia* itself has a negative impact on GE. This is mediated through relaxation in the antral portion of the stomach and the suppression of the distal contractions in the pylorus. This effect leads to reduced GE and enhances gastroparesis [39]. Additionally, this also reduces the disintegration of larger food particles which results in less chyme formation which, in effect, also aggravates the situation and reduces the motion of food from the stomach to the duodenum [40].

Yet another provocative factor for the worsening of gastroparesis in the diabetic patient are the structural changes in the stomach wall and the enteric nervous system, as long-standing diabetes leads to fibrosis in the gastric muscle layers [41], which, in effect, reduces the elasticity of the stomach and its capacity to accommodate food content. In addition to this, damage to the enteric nervous system structures within the stomach wall [42,43] further contributes to the hampered GE. Furthermore, studies have shown that individuals with diabetes also have alteration to the ultrastructure of the mucosa of the stomach which, albeit not directly related to gastroparesis, may contribute to the development of these symptoms [44,45]. Apart from the aforementioned mechanism, medications for the treatment of diabetes including GLP-1 analogs delays gastric emptying and may influence the development of gastroparesis [45].

During fasting, multiple physiological adaptations occur to conserve energy during the fasting period. For instance, GE is slowed in both animal models [46] and healthy individuals during fasting periods [47]. This would, therefore, exacerbate underlying gastroparesis if present in the individual with diabetes, causing worsened bloating and upper abdominal discomfort. In addition to this, the neurohormonal control of the stomach’s emptying is affected during fasting, such that motilin levels increase in the fasting period between meals and thereafter decrease after the ingestion of meals, whereby a delay in GE occurs [48]. Moreover, post-prandial elevation in GLP-1 and CCK causes further slowing of GE, especially in individuals with potentially underlying gastroparesis tendencies [49]. In addition to aberrancies in gastric motility, the fasting state causes biliary stasis through reduced CCK, which may increase the risk for abdominal discomfort and gallstones, with the potential for symptoms related to biliary disease overlapping those related to delayed GE [50]. Yet another mechanism involves the reduced stomach pH in the fasting state reflecting increased acidity in preparation of a meal, with an increase in pH after meal consumption [51]. These pH alterations cause further exacerbations of upper abdominal symptoms mainly through increased reflux and dyspepsia [52].

## 3. Clinical Manifestations of Gastroparesis During Ramadan

Individuals with diabetes and underlying known gastroparesis or undiagnosed gastroparesis may experience symptoms that are accentuated by prolonged periods of fasting with large meals consumed thereafter at the Iftar meal. The symptoms may include, but are not restricted to, post-prandial fullness and bloating, abdominal distension and discomfort, early satiety, and nausea which may persist for hours after ingestion [53]. In addition to this, the delayed GE may cause prolonged gastric retention of food with increased reflux into the esophagus and ensuing regurgitation symptoms [54]. These symptoms may cause the patients who—already in the non-fasting state—have difficulties maintaining sufficient caloric intake to undergo further weight loss and nutritional deficiencies if they choose to fast [55].

**Figure 2:**
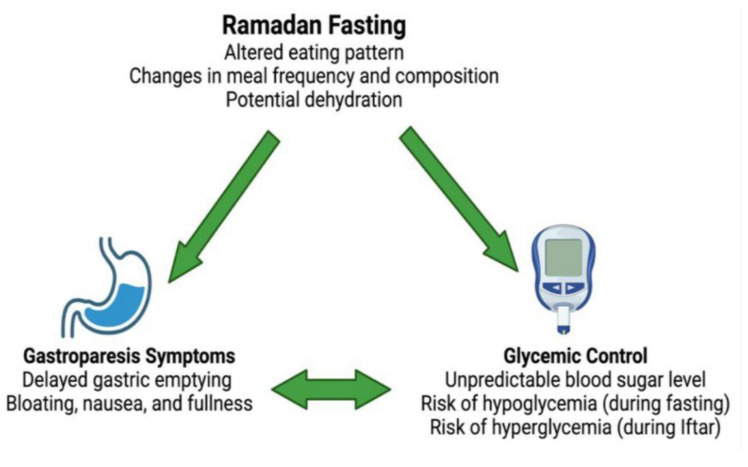
Interaction between Ramadan fasting, glycemic control, and gastroparesis symptoms. Illustration generated using BioRender™.

GE and blood glucose levels maintain a reciprocal relationship with one another [56]. As previously mentioned, hyperglycemia slows GE and hypoglycemia accelerates GE, while slowed GE causes hypoglycemia and accelerated gastric emptying causes hyperglycemia [57,58]. This is especially relevant in fasting individuals with diabetes, as when the retained stomach contents are emptied into the duodenum, a delayed glucose spike may occur with ensuing hyperglycemia most pronounced after the Iftar meal. This variability in blood glucose levels may make both the management of underlying diabetes and GE particularly challenging in Ramadan. Furthermore, the increased utilization of GLP-1 agonists in the treatment of type 2 diabetes may further hamper GE. These medications are prescribed to roughly 18% of all individuals with T2D followed in primary care in Sweden [59]. They react to slow gastric motility by both reducing antral contractions and increasing pyloric tone, thus causing a prolonged retention of food in the stomach. While this mechanism is effective in controlling blood glucose levels, patients undergoing the Ramadan fast may experience more severe gastroparesis symptoms especially if large meals at Iftar are consumed [60,61,62]. Another potential detrimental issue is the matter of fasting with regard to underlying diabetes involves the altered circadian rhythm during the Ramadan fast. This is mainly due to longer periods of sleep disruption for the Sahoor meal which is consumed just before sunrise. Such a disruption to the circadian rhythm affects underlying diabetes through impaired glycemic control, which, in effect, alters insulin sensitivity and impairs gastric emptying in the short and long terms [63]. Furthermore, older studies demonstrated delayed gastric emptying when evening meals are consumed compared to morning meals in non-diabetic individuals [64]. This further adds to the notion that gastric motility would be affected negatively when fasting in individuals already susceptible to poor GE, which fasting individuals with diabetes are considered [65]. Figure 2 summarizes the interaction between Ramadan fasting, glycemic control, and gastroparesis symptoms.

## 4. Diagnostic Approaches

In the diabetic individual planning on fasting Ramadan, a thorough evaluation of physical signs, history details, and potentially diagnostic tests may be necessary. The history details should include questions regarding post-prandial nausea, vomiting, early satiety, bloating, and abdominal pain. In addition, the correlation of these symptoms with the type and size of meals (e.g., heavy meals with fatty content) should be elicited. Indirect history signs, which may entail more widespread autonomic dysfunction, may include orthostatic hypotension, which is associated with gastroparesis in individuals with diabetes [66,67]. On physical exam, epigastric pain may be elicited upon palpation and a succussion splash may be heard using a stethoscope in the epigastrium while gently “shaking” the patient on the examination bed [68,69]. To further enhance the clinical history and examination, using the validated Gastroparesis Cardinal Symptom Index (GCSI) questionnaire [70] is warranted. The GCSI involves questions assessing symptoms centered around nausea, vomiting, post-prandial fullness, and bloating, where higher scores are significantly associated with abnormal gastric scintigraphy results [71]. This is best performed prior to the fast taking place to be able to undertake further investigations in due time before Ramadan. There are, however, certain limitations that need to be considered when using GCSI for the assessment of gastroparesis. The questionnaire is based on patients’ recall of their symptoms, and this may, therefore, introduce a source of bias. Furthermore, the correlation of GCSI to GE scintigraphy has been reported to be poor in some studies and mainly significant with regard to the correlation of early satiety and delayed GE [72,73]. In addition, symptoms associated with gastroparesis may overlap with functional dyspepsia, making symptoms reported not entirely representative of underlying delayed GE [74]. Finally, and to the best of our knowledge, the GCSI was not validated in fasting individuals, thus undermining its usage for detection of gastroparesis symptoms in that specific cohort.

To confirm clinical suspicions, quantitative measures of GE should be undertaken. Currently, the gold standard for this is gastric scintigraphy [75], which entails the consumption of a standardized meal containing a nuclear isotope whereby nuclear medicine imaging at set intervals is performed to assess the percentage retention of the meal [76]. Other imaging techniques such as wireless motility capsules or ultrasound-based GE assessment may be used [77,78]. Furthermore, indirect assessment of gastroparesis may be performed using continuous glucose monitoring (CGM), which is widely accessible nowadays through wearable devices. This is carried out through the assessment of glucose levels throughout the delay in relation to feeding. Delayed GE may be suggested if prolonged hypoglycemia followed by exaggerated post-prandial hyperglycemia is seen [79,80,81].

## 5. Management Strategies

A multidisciplinary approach to individuals with diabetes suffering from gastroparesis is required, involving cooperation between the treating physician, diabetes nurse, and potentially specialized dieticians. Multiple levels of potential intervention exist, including dietary adjustments, medications, and certain lifestyle modifications, to ensure a safe fast for individuals with diabetes who wish to fast [82]. Prior to the start of Ramadan, a detailed medical evaluation regarding gastroparesis symptoms using GCSI and detailed diabetes assessment should be performed to provide personalized recommendations tailored towards the specific patient.

*Dietary adjustments* include the intake of easily digestible items with small, frequent meals during the non-fasting portion of the day, with the gross avoidance of large but infrequent meals. It is essential to avoid fiber-rich, fatty, and heavy meals as these foods contribute to delayed GE [83]. This is especially true in the Iftar meal, where easily digestible foods such as soups and mashed vegetables should be prioritized. Focusing on nutrient-rich foods should be prioritized, with no room for high-fat foods to avoid worsened GE. In addition, adequate hydration is imperative during the non-fasting hours, as dehydration may exacerbate gastroparesis symptoms [84]. Avoiding fizzy and caffeine-rich drinks is imperative, as these items may worsen gastroparesis symptoms without directly interfering with GE [85,86].

To optimize *medical treatment*, adding frequent short-acting insulin alongside establishing the usage of CGM in patients planning to fast is important to monitor glycemic control tightly to avoid hyper- and hypoglycemic episodes, which may impair GE further [87]. Although GLP-1 analogs have been considered safe for usage in Ramadan [88], a careful assessment of the patient’s symptoms should be considered. If gastroparesis symptoms exist, a dose reduction in GLP-1 analog may be considered, with a focus on patient-specific factors, including risk for hyperglycemia and the potential for using pro-motility agents to ameliorate GLP-1 analog-related delayed GE [89]. In addition, and to optimize GE further, the usage of pro-motility agents such as metoclopramide or domperidone may be necessary. These should be taken at Iftar and Sahoor to optimize GE during meals. Antiemetics may be beneficial for controlling nausea and vomiting. For Iftar, these medications should be taken circa 30 min before proper food intake [90,91]. While these drugs are beneficial in the management of gastroparesis, their side effects may limit long-term use. Metoclopramide is primarily associated with neurological complications, including tardive dyskinesia, particularly with prolonged use, while domperidone has been linked to QT prolongation and an increased risk of cardiac arrhythmias [92]. Due to these risks, regulatory agencies such as the U.S. Food and Drug Administration (FDA) and the European Medicines Agency (EMA) have imposed restrictions on their use [93,94,95]. In addition to medical treatment, lifestyle modifications such as thorough chewing of food, avoiding the aforementioned trigger foods, and being in an upright posture for a prolonged time in the post-prandial period is essential [96].

It is important that the diabetic patient is thoroughly counseled with regard to fasting if suspicion of underlying gastroparesis exists, given the risk for symptom exacerbation during fasting. This is especially true if glycemic control is erratic during the fast or if gastroparesis symptoms worsen during the fast. Islamic teachings permit individuals with health conditions to forgo fasting for the sake of maintaining health and preserving life [97].

## 6. Research Prospects

Further research is needed to better understand the interplay between Ramadan fasting, diabetic gastroparesis, and glycemic control. While intermittent fasting has been shown to influence gastric motility through neurohormonal changes, including motilin and ghrelin fluctuations as previously mentioned, its overall impact on diabetic gastroparesis remains unclear, requiring further studies to assess these findings in fasting individuals. Large-scale prospective studies are necessary to assess the prevalence of gastroparesis in individuals with diabetes who fast, while also identifying risk factors for symptom exacerbation and whether fasting-induced changes in gastric physiology improve or worsen motility dysfunction.

Investigating the long-term effects of intermittent fasting on gastric emptying and autonomic function may provide insights into whether fasting offers protective or detrimental effects in patients with gastroparesis. Additionally, randomized controlled trials should evaluate the safety and efficacy of pharmacological interventions, particularly GLP-1 receptor agonists, in fasting individuals with diabetes who also have subclinical or overt gastroparesis. Given the close relationship between glucose fluctuations and gastric emptying, the role of CGM and digital health tools should also be explored, as glucose variations detected through CGM may serve as indirect markers for delayed gastric emptying.

## 7. Conclusions

Fasting during Ramadan can be particularly difficult for individuals with diabetes who have undiagnosed gastroparesis. Going for long hours without food, followed by consuming large meals, can worsen symptoms and make glycemic control utterly difficult to achieve. Understanding how changes in meal timing affect digestion is imperative to reduce gastroparesis symptoms. With proper diagnosis, changes in diet, lifestyle adjustments, and the right medical treatments, individuals with diabetes can better manage their condition while fasting. Healthcare professionals should offer tailored advice to help individuals fast safely while maintaining stable health. Further research—using available questionnaires and CGM data in T2D individuals—is needed to assess the proportion of fasting individuals with diabetes who develop subclinical gastroparesis symptoms. These factors, if studied, will help risk stratify individuals who are at risk of developing significant gastroparesis when undergoing the Ramadan fast.
